# Anti-D4GDI antibodies activate platelets in vitro: a possible link with thrombocytopenia in primary antiphospholipid syndrome

**DOI:** 10.1186/s13075-019-1947-2

**Published:** 2019-07-01

**Authors:** C. Barbati, L. Stefanini, T. Colasanti, E. Cipriano, A. Celia, G. Gabriele, M. Vomero, F. Ceccarelli, F. R. Spinelli, A. Finucci, M. Speziali, G. Orso, D. P. E. Margiotta, F. Conti, F. Violi, A. Afeltra, G. Valesini, C. Alessandri

**Affiliations:** 1 Department of Internal Medicine and Medical Specialties, Sapienza University of Rome, Viale del Policlinico, 155 Rome, Italy; 2 Department of Immuno-Rheumatology, Campus Bio-Medico, University of Rome, Rome, Italy

**Keywords:** Primary antiphospholipid syndrome, Platelets, D4GDI, Rho GTPases

## Abstract

**Background:**

Thrombocytopenia is a manifestation associated with primary antiphospholipid syndrome (PAPS), and many studies have stressed the leading role played by platelets in the pathogenesis of antiphospholipid syndrome (APS). Platelets are highly specialized cells, and their activation involves a series of rapid rearrangements of the actin cytoskeleton. Recently, we described the presence of autoantibodies against D4GDI (Rho GDP dissociation inhibitor beta, ARHGDIB) in the serum of a large subset of SLE patients, and we observed that anti-D4GDI antibodies activated the cytoskeleton remodeling of lymphocytes by inhibiting D4GDI and allowing the upregulation of Rho GTPases, such as Rac1. Proteomic and transcriptomic studies indicate that D4GDI is very abundant in platelets, and small GTPases of the RHO family are critical regulators of actin dynamics in platelets.

**Methods:**

We enrolled 38 PAPS patients, 15 patients carrying only antiphospholipid antibodies without clinical criteria of APS (aPL carriers) and 20 normal healthy subjects. Sera were stored at − 20 °C to perform an ELISA test to evaluate the presence of anti-D4GDI antibodies. Then, we purified autoantibodies anti-D4GDI from patient sera. These antibodies were used to conduct in vitro studies on platelet activation.

**Results:**

We identified anti-D4GDI antibodies in sera from 18/38 (47%) patients with PAPS, in sera from 2/15(13%) aPL carriers, but in no sera from normal healthy subjects. Our in vitro results showed a significant 30% increase in the activation of integrin αIIbβ3 upon stimulation of platelets from healthy donors preincubated with the antibody anti-D4GDI purified from the serum of APS patients.

**Conclusions:**

In conclusion, we show here that antibodies anti-D4GDI are present in the sera of PAPS patients and can prime platelet activation, explaining, at least in part, the pro-thrombotic state and the thrombocytopenia of PAPS patients. These findings may lead to improved diagnosis and treatment of APS.

## Background

Primary antiphospholipid syndrome (PAPS) is a condition characterized by the occurrence of thromboembolic events and/or pregnancy loss combined with presence of circulating anti-phospholipid antibodies (aPLs) [1]. Importantly, the clinical criteria include only thrombosis and pregnancy morbidity; another manifestation associated with antiphospholipid syndrome (APS), but not part of the 2006 Sydney classification criteria to define APS, is thrombocytopenia [2].

Several hypotheses were put forward to explain the causal role of antibodies in the clinical manifestation of APS, and many studies have stressed the leading role played by platelets in the pathogenesis of APS, but none is fully convincing [3]. APS is classified into primary and secondary, the latter being associated with connective tissue disease. Systemic lupus erythematosus (SLE) is the most common cause of secondary APS, and the prevalence of aPLs, either LA or aCL or anti-β2GPI, in patients with SLE is reported to be as high as 30% to 50% [4]. Recently, we described the presence of autoantibodies against D4GDI (Rho GDP dissociation inhibitor beta, ARHGDIB) in the serum of a large subset of SLE patients. Moreover, in the same patients, according to the presence or absence of serum anti-D4GDI Abs, we found a significant association between the presence of these autoantibodies and the evidence of hematologic manifestations (defined as hemolytic anemia with elevated reticulocytes, leucopenia < 4000/mm^3^ on ≥2 occasions, lymphopenia < 1500/mm^3^ on ≥2 occasions, thrombocytopenia < 100,000/mm^3^) [5]. D4GDI is a negative regulator of small GTPases of the RHO family, and we observed that anti-D4GDI antibodies activated the cytoskeleton remodeling of lymphocytes by inhibiting D4GDI and allowing the upregulation of Rho GTPases such as Rac1 [5].

Proteomic and transcriptomic studies indicate that D4GDI is very abundant in platelets [6–8]. Platelets are highly specialized cells that once generated by megakaryocytes, patrol the vasculature to ensure its integrity. Their activation involves a series of rapid rearrangements of the actin cytoskeleton, which are crucial for platelet adhesion to vascular lesions and release of bioactive molecules from their granules [9]. Small GTPases of the RHO family are critical regulators of these actin dynamics in platelets [10]. Thus, we hypothesized that autoantibodies anti-D4GDI, if present in the serum of PAPS patients, could promote platelet activation and explain, at least in part, the thrombotic events and the thrombocytopenia.

The aim of the present study was to evaluate the presence of anti-D4GDI antibodies in PAPS sera and whether they can affect platelet activation, contributing to the thrombotic events and the thrombocytopenia of PAPS patients.

## Materials and methods

### Patient and control recruitment and ELISA test

Thirty-eight PAPS patients diagnosed according to the 2006 Sydney classification criteria were enrolled from the Lupus Clinic of the Sapienza University of Rome.15 patients carrying only antiphospholipid antibodies without clinical criteria of APS (aPL carriers) and 20 normal healthy subjects (NHS) served as controls. The local ethic committee approved this study (Prot n 154/19), and participants gave their written informed consent. Sera were stored at − 20 °C to performed an ELISA test using commercial D4GDI protein.

### Purification of specific autoantibodies from patient sera

Recombinant D4GDI (50 μg) was spotted onto a nitrocellulose filter and incubated with sera from APS patients that had OD > 0.86 by ELISA. The antibodies were eluted with 100 mM glycine, pH 2.5, immediately neutralized with 1 M Tris HCl, pH 8, and dialyzed against PBS.

To exclude variations in binding reactivity after the purification process, purified anti-D4GDI antibodies were tested by ELISA [5].

### Platelet preparation and in vitro activation

Venous blood was drawn in trisodium citrate (3.8%, 1/10 (*v*:*v*)) from NHS (*n* = 5) who had fasted for at least 12 h. To wash platelets, blood was immediately centrifuged for 15 min at 180 g at room temperature, and platelet-rich plasma (PRP) was separated. To avoid leukocyte contamination, only the top 75% of the PRP was collected. PRP was centrifuged twice in the presence of prostaglandin I_2_ for 10 min at 300*g* and resuspended in Tyrode’s buffer containing mmol/L (137 NaCl, 0.3 Na_2_HPO_4_, 2 KCl, 12 NaHCO_3_, 5 N-2-hydroxyethylpiperazine-N′-2-ethanesulfonic acid, 5 glucose) pH 7.3 containing 0.35% BSA (fraction V, Sigma-Aldrich) to a final concentration of 10^8^ platelets/ml. Washed platelets were then incubated at 37 °C with 20 μg/ml of the purified antibody anti-D4GDI or with the same volume of buffer (PBS).

For determination of integrin αIIbβ3 activation, a well-established marker of platelet activation and adhesion, 20 μl of pre-treated platelets was stimulated with adenosine diphosphate (ADP, 10 μM; Sigma-Aldrich, Saint Louis, USA) or not (no stimulation) for 10 min in the presence of 1 mM CaCl_2_ and 5 μg/ml PAC1-FITC (BD Biosciences, cat # 340507), an antibody directed towards the activated form of human αIIbβ3 [11]. Following stimulation, the samples were diluted with 1 ml of PBS and analyzed immediately with a BD Accuri™ C6 Plus flow cytometer. Data is shown as % of control, considering the mean fluorescence intensity of the activated control as 100% (mean ± SD).

For determination of integrin αIIbβ3 activation in real time, washed platelets pre-treated with 20 μg/ml of anti-D4GDI or buffer were further diluted to 5 × 10^6^ platelets/ml. After establishing a baseline with unlabeled platelets, 5 μg/ml PAC1-FITC and 25 μM ADP were added simultaneously in an equal volume of modified Tyrode’s buffer to allow efficient mixing. PAC1-FITC binding was recorded continuously for 150 s with a BD Accuri™ C6 Plus flow cytometer [12].

### Statistical analysis

Normal distribution of variables was assessed using the Kolmogorov-Smirnov test. Statistical analysis was performed using the program GraphPad Prism Version 6 (GraphPad Software, San Diego, CA, USA). The Mann–Whitney unpaired test or Student’s *T* test were used to compare quantitative variables in different groups. Statistical correlation was examined using Spearman’s rank correlation coefficient. Values of *p* < 0.05 were considered statistically significant.

## Results

### Identification of anti-D4GDI antibodies in patients’ sera and association with thrombocytopenia

**Fig. 1:**
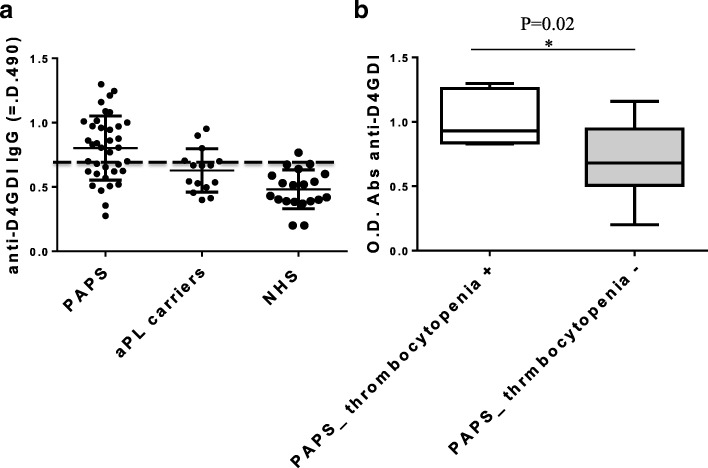
Evaluation of the specific antibody titer for D4GDI in sera from PAPS patients and normal healthy donors and association of anti-D4GDI antibody titer and thrombocytopenia in PAPS patients. **a** The image shows the cut-off calculated on normal healthy donors (OD 490 = 0.86); 18 PAPS patients out of 38 (47%) have values higher than the cut-off and therefore considered positive. Only 2 aPL carriers out of 15 have values higher than the cut-off. **b** The boxes show how PAPS patients with thrombocytopenia have a higher serum titer of anti-D4GDI antibodies

We identified anti-D4GDI antibodies in sera from 18/38 (47%) patients with PAPS, in sera from 2/15 (13%) aPL carriers, but in no sera from normal healthy subjects (Fig. 1a). Dividing the patients with APS according to the presence or absence of thrombocytopenia, we found a significant association between this hematologic manifestation and a higher titer of anti-D4GDI antibodies (Fig. 1b). There was no significant correlation with other clinical features and presence of aPLs antibodies (data not shown).

### Role of anti-D4GDI antibodies in platelet activation

**Fig. 2:**
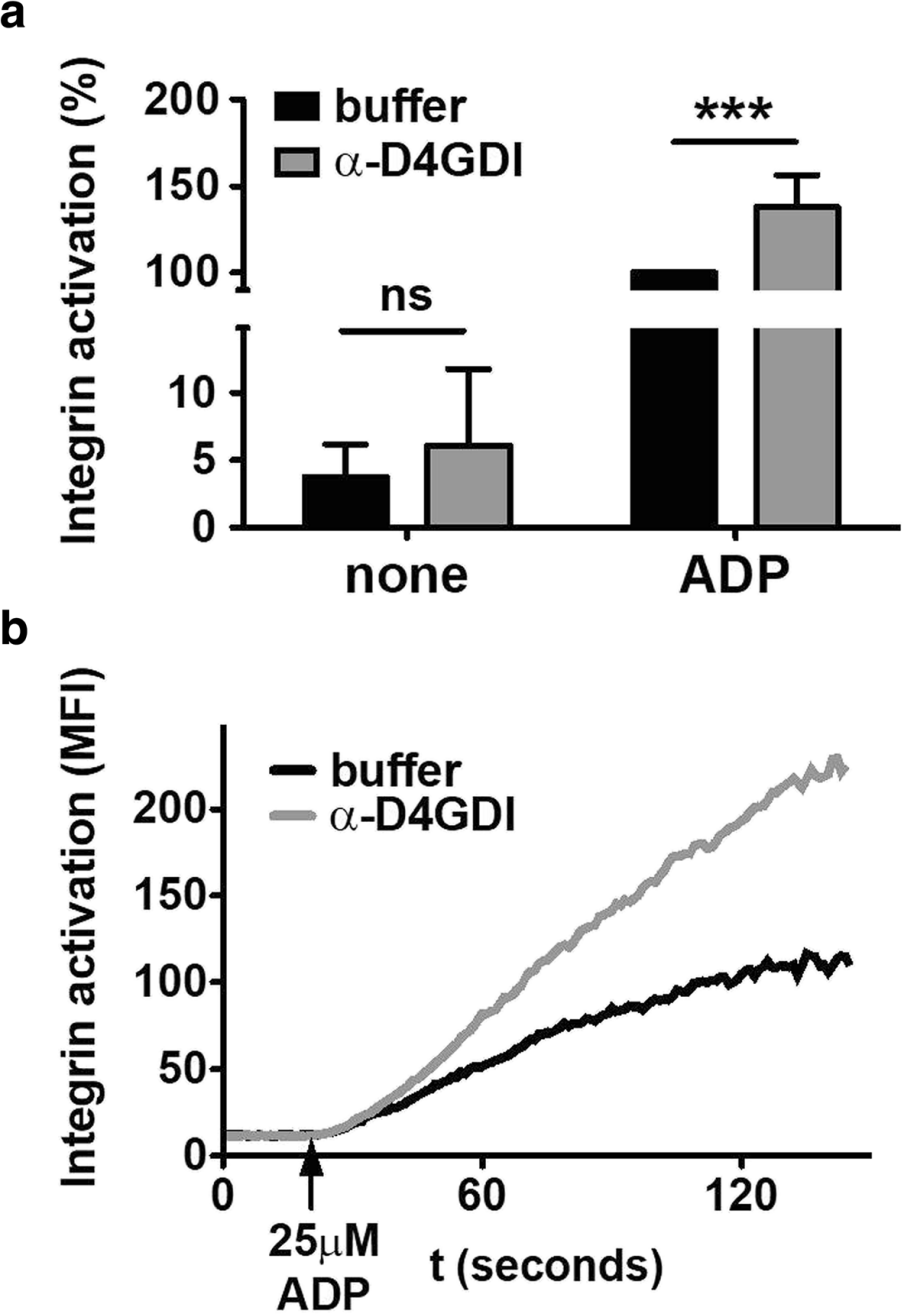
Platelet activation in the presence of anti-D4GDI antibodies from the sera of PAPS patients. Preincubation of platelets from healthy donors with 20 μg/ml of anti-D4GDI antibodies, purified from the sera of PAPS patients, significantly increases the **a** total % (*n* = 5) and the **b** kinetic rate (representative image, *n* = 5) of adenosine diphosphate (ADP)-induced integrin activation (gray line and bar), compared to controls with buffer only (black line and bar). In the absence of agonist stimulation (none), there is a similar trend but is not statistically significant

Thrombocytopenia can be due to reduced platelet production at the level of megakaryocytes or to increased platelet consumption. We hypothesize that the antibody anti-D4GDI has a priming effect on the activation of platelets and thereby contributes to the thrombocytopenia by increasing the consumption of activated platelets that freely circulate in the bloodstream. To test our hypothesis, we measured integrin αIIbβ3 activation in basal and ADP stimulated conditions in the presence or absence of anti-D4GDI. Our results show (Fig. 2 panel a) a significant 30% increase in the activation of integrin αIIbβ3 upon stimulation of platelets from healthy donors preincubated with the antibody anti-D4GDI purified from the serum of APS patients. The activating effect of the antibody is also detectable in basal (non-stimulated) conditions, but it is not statistically significant. Interestingly the antibody does not only increase the overall integrin activation but also the rate/speed of integrin activation (panel b). These results suggest that the presence of the antibody anti-D4GDI in circulation primes platelets for activation and promotes their rapid switch from an anti-adhesive to a pro-adhesive state when other stimuli, such as ADP leaking from red blood cells, are present.

## Discussion

The main findings of this study are (1) that we detect anti-D4GDI antibodies in 47% of PAPS patients, (2) that a higher titer of anti-D4GDI antibodies is significantly associated with thrombocytopenia, and that (3) the antibodies anti-D4GDI isolated from the serum of APS patients promote agonist-induced platelet activation in vitro.

D4GDI belongs to the family of RhoGDI or GDP-dissociation inhibitors for the Rho/Rac family of small G proteins [10, 11]. Like other G proteins, RHO GTPases cycle between an inactive GDP-bound form and an active GTP-form. GDP-dissociation inhibitors stabilize the GDP-bound form thus inhibiting RHO-GTPase function. It is well known that D4GDI is highly expressed in hematopoietic tissues. However, the intracellular regulatory function of D4GDI is still poorly understood. The only known fact is that D4GDI, like several other surface molecules [12], is embedded into lipid rafts, subdomains of the plasma membrane that provide a scaffold for signal transduction proteins. In our previous study, we characterized D4GDI as a peripheral blood lymphocyte antigen recognized by serum autoantibodies from a 46% of patients with SLE [5]. The presence of these autoantibodies appeared associated with (i) increased levels of active (GTP-bound) Rac1 and Rho small GTPases and (ii) increased Rho GTPases functional responses, i.e., actin filament polymerization, in lymphocytes [5].

Recent proteomic and transcriptomic studies indicate that D4GDI is highly expressed in platelets [6–8]. Consistently with the inhibitory role of D4GDI and with our previous observations in lymphocytes, when we incubate platelets of healthy donors with anti-D4GDI antibodies, we observe an increase in the rate and overall activation of integrin αIIbβ3, the integrin that supports platelet-platelet aggregation [9]. Thus, it is plausible that this antibody contributes to the pro-thrombotic state of PAPS patients. Since the effect of anti-D4GDI antibodies on platelet activation is statistically significant upon agonist stimulation but not in resting non-stimulated conditions, we presume that these antibodies do not have a direct stimulatory effect but only a priming effect by releasing one of the brakes that keep platelets inactive in circulation. Thus, allowing their rapid switch from an anti-adhesive to a pro-adhesive state when other stimuli, such as ADP leaking from red blood cells, are present.

Thrombocytopenia is the most common hematological feature of patients with antiphospholipid syndrome [2], and we show here an association between the presence of anti-D4GDI antibodies and low platelet count. Thrombocytopenia can be due to reduced platelet production at the level of megakaryocytes or to increased platelet consumption. Since anti-D4GDI primes platelet activation in vitro and PAPS are by definition pro-thrombotic, we believe that the low platelet count is mainly due to an increased clearance of platelets that are circulating in a pre-activated state. However, since RHO GTPases are also important for megakaryocyte function and pro-platelet release, we may not exclude that the anti-D4GDI antibodies also affect platelet production in the bone marrow.

## Conclusions

In conclusion, we show here that antibodies anti-D4GDI are present in the sera of PAPS patients and can prime platelet activation, explaining, at least in part, the pro-thrombotic state and the thrombocytopenia of PAPS patients. These findings may lead to improved diagnosis and treatment of APS.

## Data Availability

Datasets analyzed during the current study are available from the corresponding author on reasonable request.

## Abbreviations

aPLs: Anti-phospholipid antibodies
D4GDI: Rho GDP dissociation inhibitor beta
NHS: Normal healthy subjects
PAPS: Primary antiphospholipid syndrome
PRP: Platelet-rich plasma
SLE: Systemic lupus erythematosus
